# Comparison of Muscle Strength, Aerobic Capacity and Body Composition between Healthy Adolescents and Those Living with HIV: A Systematic Review and Meta-Analysis

**DOI:** 10.3390/ijerph18115675

**Published:** 2021-05-26

**Authors:** Rafaela Catherine da Silva Cunha de Medeiros, Isis Kelly dos Santos, Anna Luiza Vasconcelos de Oliveira, Carlos Jean Damasceno de Goes, Jason Azevedo de Medeiros, Tatiane Andreza Lima da Silva, Juliany de Souza Araujo, Phelipe Wilde de Alcântara Varela, Ricardo Ney Cobucci, Breno Guilherme de Araújo Tinoco Cabral, Paulo Moreira Silva Dantas

**Affiliations:** 1 Graduate Program in Health Sciences, Federal University of Rio Grande do Norte, Natal 59078-970, Brazil; isisk2@hotmail.com (I.K.d.S.); jason.medeiros1@hotmail.com (J.A.d.M.); tatianeandreza@yahoo.com.br (T.A.L.d.S.); brenotcabral@gmail.com (B.G.d.A.T.C.); pgdantas@ufrnet.br (P.M.S.D.); 2 Department of Nutrition, Federal University of Rio Grande do Norte, Natal 59078-970, Brazil; vasconcelosannaluiza@gmail.com; 3 Department of Physical Activity, Federal University of Rio Grande do Norte, Natal 59078-970, Brazil; carlosjeangoes@hotmail.com (C.J.D.d.G.); julianysa@hotmail.com (J.d.S.A.); phelipewilde@ufrn.edu.br (P.W.d.A.V.); 4 Biotechnology Graduate Program, Potiguar University of Rio Grande do Norte, Natal 59078-970, Brazil; rncobucci@unp.br

**Keywords:** physical fitness, cardiorespiratory fitness, child health, adolescents, human immunodeficiency virus

## Abstract

*Background*: The adverse effects of antiretroviral therapy associated with complications generated by human immunodeficiency virus (HIV) promote impairments in physical fitness in adolescents. *Objective*: To analyze the aerobic capacity, muscle strength, and body composition of adolescents living with HIV compared with a healthy population of the same age. *Methods*: Searches were performed in the MEDLINE, Embase, Web of Science, Scopus and SportDiscus databases until September 2019 and updated in April 2020. Eligibility Criteria: adolescents of both sexes in the age group from 10 to 19 years; living with HIV; cross-sectional, case–control, cohort studies; comparing with a healthy population. Mean differences and 95% Confidence intervals (CIs) were calculated using RevMan (software for systematic reviews). *Results*: Five articles were included, involving 197 adolescents living with HIV (16 to 18 years) and 185 without infection (13 to 18 years), with the sample in each study ranging from 15 to 65 adolescents. Aerobic capacity and muscle strength were reduced in adolescents with HIV, and body mass index was also significantly lower in this group. *Conclusion*: Adolescents living with HIV have impaired cardiorespiratory fitness, muscle strength, and body composition when compared to their uninfected peers. However, this systematic review provides limited evidence on the differences between the physical fitness outcomes of adolescents living with HIV compared to healthy adolescents.

## 1. Introduction

Globally, data indicate that the population of adolescents living with human immunodeficiency virus (HIV) is continuing to grow. It is estimated that there are about 2.1 million children and adolescents aged 10–19 years [1,2] who need to live longer with the virus, and have worse HIV-related outcomes [3].

The use of antiretroviral therapy (ART) aims to suppress viral load and significantly restore the immune system by preserving T cells that express cluster determinant 4 (CD4 T) lymphocytes, the main target cell of the virus, reducing the incidence of infectious complications and the risk of death [3]. In addition, ART contributes to disease control, offers a significant increase in life expectancy, and also improves the quality of life of these individuals [4].

Despite the benefits of ART, adverse effects are reported, including metabolic complications and structural abnormalities, which promote increased risk of growth and developmental disruption, resulting in significant changes in parameters of Physical Fitness (PF), such as muscle changes and abnormalities in body composition [5,6,7,8,9].

It is relevant to mention that these changes may favor a decrease in health-related physical aptitudes and increase the risk of developing cardiovascular diseases in children and adolescents living with HIV compared to the uninfected population in the same age group [6,10,11,12,13]. In the literature, the importance and benefits of physical activity for the health of this population are well established; however, the physical fitness of adolescents living with HIV needs to be discussed more [14]. Adolescents with HIV are reported to have lower overall scores for habitual physical activity, with shorter times for physical activity after school. Reinforcing these issues, children and adolescents living with chronic conditions other than HIV also have restrictions and inadequate levels of physical activity and sports practice, all of which predispose the individual to negative health factors and low physical fitness due to the sedentary lifestyle adopted because of various conditioning factors [11,15,16,17].

Lower values for physical fitness-related abilities are observed in children and adolescents living with HIV, such as agility, flexibility, strength, and muscular endurance [18,19,20]. Even with certain evidence available, there are few studies in these populations that evaluate all the parameters related to health and physical performance. Some studies have been published on this topic; however, only one systematic review has been published comparing individuals living with HIV with healthy controls, with emphasis on only aerobic capacity, without highlighting information about body composition, especially in the pediatric population [21]. Thus, researching how the retrovirus and ART interferes with the physical fitness of adolescents can help health professionals find the best forms of prevention and treatment for this population so as to avoid medium- and long-term clinical repercussions. Thus, the present systematic review compared the aerobic capacity, muscle strength, and body composition of adolescents living with HIV with the population without infection.

## 2. Methods

### 2.1. Protocol and Registration

This systematic review was prepared in accordance with the Preferred Item guidelines for Systematic Reviews and Meta-Analyses (PRISMA) [22]. The protocol for this review was registered in the PROSPERO database under number CRD42019140955.

### 2.2. Eligibility Criteria

The studies eligible for this systematic review met the following criteria: (a) conducted with adolescents of both sexes in the age group from 10 to 19 years of age, which is the World Health Organization (WHO) definition of adolescence; (b) adolescents living with HIV; (c) cross-sectional, case–control, cohort studies comparing a healthy population of the same age group; (d) studies which reported at least one assessment of physical fitness parameters such as: muscle strength or endurance (assessed in either field or laboratory tests) and flexibility; (e) no restrictions on year and language.

In addition, the forms of assessment used to measure body composition in the studies were: dual energy X-ray absorptiometry (DXA) and anthropometry; cardiorespiratory function: modified Balke and Bruce protocol; agility: shuttle Ru test; flexibility: the modified sit and reach test; Strength and muscular endurance: 1-Repetition Maximum—1RM, dynamometry, and tests of vertical thrust.

Review articles, study validations, conference abstracts, monographs, dissertations, theses, comments, brief reports, and studies conducted with special populations such as children or adolescents with developmental disabilities and cognitive delays were excluded.

### 2.3. Search Strategy and Study Selection

The search was defined based on the PECOS strategy (Patients, Exposure, Comparison, Outcome, Type of study). To identify studies in the databases, the descriptors, keywords, and their synonyms searched in the Medical Subject Headings (MeSH) were used. The search strategies used were: (physical fitness OR physical endurance OR muscle strength OR physical conditioning OR cardiorespiratory fitness OR aerobic capacity OR flexibility OR pliability) AND (human immunodeficiency virus OR HIV) AND (child OR children OR adolescents OR teens OR teenagers) (Supplementary 1 in Supplementary Material). The search strategies mentioned were systematically used in the following databases: MEDLINE (via PubMed), Embase (via Ovid), Web of Science, Scopus, and SportDiscus. The gray literature database was Open Grey (http://www.opengrey.eu/, accessed on 5 September 2020). The literature searches were performed until the date of September 2019 and were updated in April 2020. The search methods were adapted to other databases; all the references from the articles were analyzed with the perspective of including other referenced studies.

After the search phase, Rayyan QRCI [23] software (Qatar Computing Research Institute (Data Analytics), Doha, Qatar) was used to track duplicates and read studies. Four independent reviewers (RCSCM, IKS, CJDG, and ALVO) read the titles and abstracts of the articles. Subsequently, the full texts of the documents for analysis were obtained. In the case of conflicting study analysis, a third reviewer was consulted to clarify doubts (PMSD). Inter-reviewer agreement was calculated using the Kappa index for each phase of article selection (kappa = 0.74).

### 2.4. Data Extraction

After final analysis, two reviewers (CJDG and ALVO) extracted the following data from all the eligible articles: (a) year of publication, (b) study design, (c) location, (d) participant characteristics (age, gender), (e) sample size, (f) medication use, (g) main results and instruments used (components of physical aptitude), (h) control variables (sociodemographic, clinical and physical activity level variables), and (i) main results.

### 2.5. Quality Assessment

To assess the methodological quality of the included studies, we used a scale based on the NIH Quality Assessment Tool for Observational Cohort and Cross-Sectional Studies (https://www.nhlbi.nih.gov/health-topics/study-quality-assessment-tools, accessed on 25 October 2020) which contains three domains (selection bias, measurement, result and reporting bias) with 14 items. This tool is based on other studies with the same population that used the Cochrane criteria for observational studies [24,25]. In this sense, the scale determines how each study addressed questions related to: (1) Research question; (2) Study population; (3) Appropriate sample size; (4) Groups recruited from the same population and uniform eligibility criteria; (5) Justification of sample size; (6) Presentation of results; (7) Deadline to analyze effect; (8) Differences between exposure levels of interest; (9) Exposure and assessment measures; (10) Repeated exposure assessment; (11) Outcome measures (12) Blindness of outcome evaluators; (13) Follow-up rate; and (14) Statistical analysis (Supplementary 2 in Supplementary Material). For the final analysis of the quality scores of the 14 items, the values are divided into 14 points, where values below 7 points indicate high risk, between 7 and 10—moderate risk, and above 10 points—low risk of bias. Two reviewers (RCSCM and IKS) independently assessed the studies and rated each criterion as “yes”, “no”, or “other (not clear; uncertain)”. Any conflict in the assessment was independently resolved by a third reviewer (RNC).

### 2.6. Quantitative Analyses or Statistical Assessment

Data on mean and standard deviation (SD) of weight, height, body mass index (BMI), muscle strength, and peak oxygen consumption (VO_2_ max) were extracted for the purpose of calculating differences between groups (Table 1). When necessary, SD values were extracted or calculated using available data (e.g., standard error or confidence intervals (CIs)) or information presented in a table. We contacted some authors in order to collect data that were not reported in the study.

Results were grouped according to the measurement type and compared HIV-infected patients with matched healthy controls younger than 18 years of age. Analyses were conducted using Review Manager Version 5.3 (The Cochrane collaboration, Copenhagen, Denmark).

## 3. Results

### 3.1. Study Selection

**Figure 1:**
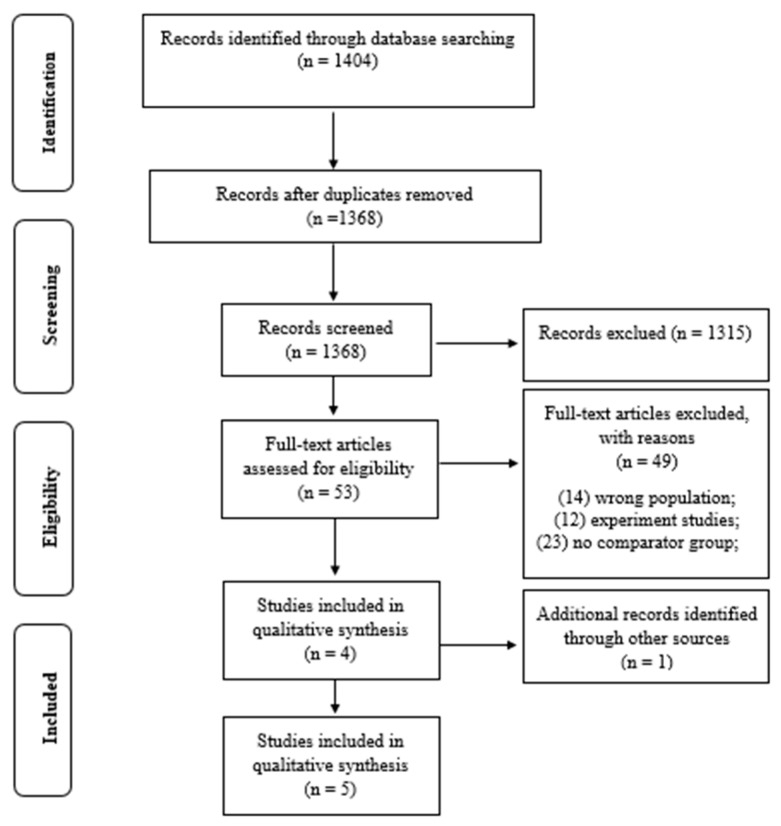
Search and selection of studies for systematic review according to PRISMA.

The initial search identified a total of 1404 potential articles; after the removal of 36 duplicate studies, 1368 studies remained. Of these, 1315 studies did not meet the criteria after reading the titles and abstracts. A total of 53 studies were examined, of which 49 were excluded, leaving only 4 studies to be included in the systematic review. After tracing the references, only one additional study was identified, resulting in a total of five articles (Figure 1). The excluded studies are presented in the Supplementary Material in Table S1.

### 3.2. Characteristics of Included Studies

Table 1 and Table 2 summarize the study characteristics and population, methods used to test muscle strength, aerobic capacity, and body composition, as well as analysis of the methodological quality. After the screening process, a total of five studies from three different countries were included in this review; two compared infected group muscle strength and control, three showed cardiorespiratory capacity, and four included body composition among adolescents living with HIV and healthy controls.

The number of participants in the studies ranged from 15 to 65 adolescents, totaling 197 in the retrovirus group and 185 in the healthy group. From analyzing the age groups of the studies, it was found that the age ranged from 16 to 18 years in the first group and 13 to 18 years in adolescents without infection. The five studies included both male and female outpatients diagnosed with HIV; 179 adolescent perinatally infected with HIV; and four studies using ART or Highly Active Antiretroviral Therapy (HAART). 

Table 3 presents the differences in physical fitness variables between the HIV group and healthy group, demonstrating an estimate of effect on all variables analyzed.

#### 3.2.1. CD4/Viral Load/ARV or HAART

One study report that sex, ART, and economic level explained 33% of the sum of skin folds in the HIV + group. The body composition of patients living with HIV is usually modified in the interaction between the virus, HAART, and the host. Clinical and sociodemographic variables can also express a stronger relationship with body composition [11].

In one study, all subjects except one were on a HAART medication regimen, which included nucleoside analogue medication [12].

HIV infection was associated with a decrease in aerobic fitness. This decrease was bigger in untreated patients compared to PI-based HAART patients [27]. HAART was independently associated with lower exercise capacity in HIV-infected children and greater exposure to Nucleoside Reverse Transcriptase Inhibitors (NRTIs) and Protease Inhibitors (PIs) affected the VO_2_ peak [6].

#### 3.2.2. Muscle Strength

Muscle strength was evaluated in only two studies [6,26], in which one study used the maximum repetition evaluation test, while the other study used the isokinetic strength test to quantify lower limb muscle strength. The studies that evaluated strength had a total of 111 participants (60 with HIV and 51 healthy controls). The results found in one study show that muscle strength was significantly lower in adolescents living with HIV; in another study, the difference between groups was detected in the muscle strength test in lower body strength. Due to the lack of data (mean and SD), it was not possible to examine the average difference between studies.

#### 3.2.3. Aerobic Capacity

Aerobic capacity was assessed in three studies using the cardiopulmonary exercise test with a treadmill and treadmill cycle, in which VO_2_ peak was evaluated as a result [6,12,28]. A total of 241 participants (125 HIV-infected and 116 healthy controls) were included in these three studies. The results of the three studies showed that aerobic capacity was significantly lower in adolescents living with HIV (−6.11; 95% CI −7.71, −4.52, *p* = 0.001) compared with adolescents in the healthy group.

#### 3.2.4. Body Composition

Body composition was assessed in four of the five included studies. Of these, three used the anthropometric method and only one study used the DXA method, which is the gold standard for this evaluation [6,11,26,28]. The studies included 382 participants (197 infected with HIV and 185 healthy controls). There was a significant difference in body mass index of (0.25; 95% CI 0.17, 0.33, *p* = 0.001) for participants in the HIV group compared with the healthy control group.

## 4. Discussion

This systematic review reveals as a main result that adolescents living with HIV have impaired aerobic capacity and muscle strength, as well as a predominance of lower weight and height when compared to their peers without infection. However, it provides limited evidence on the differences between the physical fitness outcomes of adolescents living with HIV compared to healthy adolescents.

Concerning the difference in body composition of patients living with HIV, there is an association with the interaction between virus, HAART, and host [29]. The change in phenotype includes body fat distribution abnormalities, which represent increased risk for premature cardiovascular diseases and accumulation of visceral fat, which at high levels is associated with chronic inflammation and metabolic disorders [5]. The use of HAART does not fully reverse the effects of HIV child growth [30]. However, a systematic review that addressed the influence of antiretroviral therapy on the growth pattern in children and adolescents living with HIV indicated that ART may influence weight and height development, indicating that the earlier the diagnosis of infection and, concomitantly, beginning of the use of combination antiretroviral therapy, the lower the impairment of growth compared to healthy children [29].

There are also alterations in anthropometric variables, with a difference in weight, height, and consequently, BMI in children and adolescents living with HIV compared to adolescents without infection. Such phenotype change can be observed through the involvement of height–weight development (HWD), which can occur in the first months of life. The onset of the pubertal stage in HIV-infected adolescents is late and the magnitude of this delay is directly related to the severity of the clinical manifestations of the infection, generating consequences such as lipodystrophies and chronic inflammation [30].

Early clinical involvement in vertically acquired HIV and the long duration of ART use may interfere with health-related parameters such as growth, nutritional status, and body composition in HIV-infected adolescents [31,32,33]. However, high levels of physical inactivity may justify losses in physical fitness parameters such as body composition and muscle strength [31] because this pathological state may cause hypoactivity, thus reducing functional capacity [15]. There are improvements in these variables among people with HIV who are classified as “active”, since the practice of physical activity helps in the development of beneficial and welfare factors for children and young people and has positive effects on metabolic, morphological, psychological, and functional parameters [26,34].

Regarding the lower values for aerobic capacity of adolescents living with HIV, these results reinforce asymptomatic abnormalities in the cardiovascular structure, as evaluations indicate systolic dysfunction in the left ventricle (LV), LV hypertrophy, and left atrial dilation in patients using ART. Moreover, diastolic dysfunction is common in long-term survivors of HIV infection, and rates of congenital cardiovascular malformations vary in HIV-infected children [35]. This explanation is due to the fact that normal development, for example of the human lung, begins in the womb, but continues in postnatal life until adolescence. Thus, factors that impair lung growth (e.g., HIV infection and ART use) may have a considerable impact on aerobic capacity [36].

Aerobic capacity is one of the widely used measures of physical fitness, and is considered a measurement of general physical health. Therefore, these results are worrisome and warn of the need and importance of adherence of children and adolescents to interventions that prioritize the performance of daily physical activity, whether at school, leisure, or home [37]. Long exposure to ART, the virus, and reduced level of physical activity may contribute to the development of low-grade chronic inflammation associated with increased likelihood of cardiovascular disease [11,16,32].

The results on muscle strength in the present study are limited, and do not allow any evidence of significant differences between groups, but children with HIV infection show less body strength than healthy peers. Studies indicated that the muscle strength of adolescents living with HIV was impaired compared to the healthy group [6,27]. These results indicate the development of clinical implications in general, precisely because most activities in the daily life of children and adolescents, such as games, play, and sports activities, are usually short term and high intensity, that is, they require anaerobic metabolism [38]. In addition, the improvement of this capacity occurs as the muscle mass increases, being linked to maturation and hormonal modifications [16].

It is relevant to mention that, in general, there is a physiological explanation for children and adolescents at the beginning of pubescence presenting an underdeveloped anaerobic system. For example, morphological characteristics play an important role in the use of energetic substrates metabolized by the anaerobic system; thus, children at the onset of pubescence tend to have lower stored endogenous glycogen content due to a higher predominance of type I muscle fibers (slow-twitch oxidants), which are mostly stimulated in long-term, low-intensity activities using free fatty acids for energy. However, according to growth and development, the production of androgenic and estrogenic hormones participates in the transition from type I muscle fibers to type II (glycolytic) fibers, causing adolescents in their mid and late pubescence to better metabolize carbohydrates and perform better in short-term, high-intensity activities [37]. In the pediatric population with HIV, these characteristics may be further impaired as aerobic metabolism is already impaired due to mitochondrial toxicity, which is present due to direct actions of the virus and drug therapy. Thus, this population ends up with both aerobic and anaerobic metabolic deficiency [18].

In this sense, monitoring the growth and developmental factors of children with HIV are key indicators for analyzing treatment responses through the use of ART (after months of exposure), which is known to play a role in improving viral and immunological markers, as well as substantially reducing morbidity and mortality in children and adolescents living with HIV, and assisting in the recovery of weight and height scores, especially when it has been started early in asymptomatic children without weight–height impairment [18,39].

It is interesting to consider that this review has some limitations, since despite the rigorous criteria adopted for the inclusion of the studies, none of the included studies showed a good quality of methodology according to the evaluation. In addition, the use of different methods of assessment for cardiorespiratory fitness, muscle strength, and body composition variables, significant differences in the mean age of participants in the studied groups, lack of standardization of physical capacity assessment, and the small sample size in the studies included in the review prevent the results from being extrapolated to all adolescents living with the virus.

This systematic review is the first to analyze body composition, muscle strength, and aerobic capacity among adolescents living with HIV compared with healthy adolescents as most HIV studies focus on adult health. Therefore, there are limitations that need to be highlighted, such as the lack of randomized clinical trials (RCT) involving children and adolescents living with HIV, the inclusion of only cross-sectional studies, and the small sample included. We suggest that future studies may be directed to conducting RCT with standardized tests and inserting interventions with physical exercise or lifestyle changes within the school or home context, making it possible to analyze the effects of these interventions on the improvement of these parameters in this population, as well as the insertion of analyses on the duration of the disease, the use of ART, and the specificity of the drugs used.

## 5. Conclusions

Adolescents living with HIV have impaired cardiorespiratory fitness, muscle strength, and body composition when compared to their uninfected peers. However, this systematic review provides limited evidence due to the small samples of available studies, the difference in measurement protocol, and high risks of bias in the studies. Randomized clinical trials and observational studies with standardized protocols are necessary to confirm the main results of this review.

## Figures and Tables

**Table 1:** Characteristics of the studies included in the review.

Study Characteristics				HIV Group				Control Group	
Study	Type of Study	Country	Exercise Status	N Analyzed; Age; Sex;	HIV-infected	N Use ART/HAART	Socioeconomic Status	N Analyzed Age Sex	Socioeconomic Status
Cade et al., 2002 [12]	Cross-sectional	USA	No exercise program	N = 15	15 horizontally	14 ART 1 HAART	NA	N = 15	NA
				18.3 (0.3) yrs				18.3 (0.3) yrs	
				11 W				11 W	
Ramos et al., 2012 [26]	Cross-sectional	Puerto Rico	No exercise program	N = 15	15 vertically	NA	Low economic status	N = 15	Low economic status
				11 yrs				11 yrs	
				7 M				7 M	
Somarriba et al., 2013 [6]	Cross-sectional	USA	No exercise program	N = 45	42 vertically 3 horizontally	37 ARV 8 HAART	NA	N = 36	NA
				16.1 (2.66) yrs				13,5 (3.01) yrs	
				53% M				61% M	
Lima et al., 2017 [27]	Cross-sectional	Brazil	No exercise program	N = 65	65 vertically	11 Not treated; 15 Non-PI-HAART 39-PI-HAART	75% low and middle income	N = 65	74% low and middle income
				12.2 (2.1) yrs				12.1 (1.8) yrs	
				46.2% M				46.2% M	
Martins et al., 2017 [11]	Cross-sectional	Brazil	General physical activity	N = 57	57 vertically	57 ARV	Low economic status	N = 54 12.8 (2.26) yrs	Low economic status
				12.9 (1.53) yrs					
				57.1% W					

N—number; yrs—years; M—men; W—women; NA—not assessed; HAART—highly active antiretroviral therapy; USA—United States of America.

**Table 2:** Cont. characteristics of the studies included in the review.

Study	Strength Measure	Aerobic Capacity Measure	Body Composition	Quality Rating *	
Cade et al., 2002 [12]	NA	VO_2_ peak (peak exercise tests—treadmill)	NA	7	Fair
Ramos et al., 2012 [26]	Isokinetic dynamometer	NA	Anthropometry	5	Poor
Somarriba et al., 2013 [6]	1RM	VO_2_ peak (mL/kg/min)	DXA	5	Poor
Lima et al., 2017 [27]	NA	VO_2_ peak—cycle ergometer test	Anthropometry	6	Poor
Martins et al., 2017 [11]	NA	NA	Anthropometry	5	Poor

1RM—one-repetition maximum assessment test; NA—not assessed; VO_2_ Peak—peak oxygen consumption; DXA—dual X-ray absorptiometry. * Quality Assessment Tool for Observational Cohort and Cross-Sectional Studies.

**Table 3:** Difference in physical fitness variables between HIV group vs. healthy group.

Outcomes or Subgroup	Studies	Participants	Statistical Method	Effect Estimate, 95% CI	*p* Value
Weight	5	382	Mean Difference (IV, Fixed, 95% CI)	−4.53 [−5.18, −3.88]	0.0001
Height	5	382	Mean Difference (IV, Fixed, 95% CI)	−7.23 [−7.64, −6.83]	0.0001
BMI	5	382	Mean Difference (IV, Fixed, 95% CI)	0.25 [0.17, 0.33]	0.0001
VO_2_	3	241	Mean Difference (IV, Fixed, 95% CI)	−6.11 [−7.71, −4.52]	0.0001

VO_2_ Peak—peak oxygen consumption; BMI—body mass index.

## Data Availability

The data that support the findings of this study are available from the corresponding author, P.M.S.D, upon reasonable request.

## Supplementary Materials

The following are available online at https://www.mdpi.com/article/10.3390/ijerph18115675/s1.

## Abbreviations

**HIV**	Human immunodeficiency virus
**AIDS**	Acquired Immunodeficiency Syndrome
**ART**	Antiretroviral Therapy
**T CD4**	CD4 cells—positive T-Lymphocytes
**PF**	Physical Fitness
**WHO**	World Health Organization
**DXA**	Dual energy X-ray absorptiometry
**1RM**	Repetition Maximum
**VO_2_**	peak oxygen consumption
**MeSH**	Medical Subject Headings
**BMI**	body mass index
**PRISMA-P**	Preferred Reporting Items for Systematic Reviews and Meta-Analysis Protocol
**LV**	left ventricle
**HWD**	height–weight development
