# Supplementary material for: Comparison of Muscle Strength, Aerobic Capacity and Body Composition between Healthy Adolescents and Those Living with HIV: A Systematic Review and Meta-Analysis
Source: Int J Environ Res Public Health. 2021 May 26;18(11):5675. doi: 10.3390/ijerph18115675 (PMC8198095; doi:10.3390/ijerph18115675)
Supplement: Supplementary file 1 [file ijerph-18-05675-s001.zip › Supplement 1-Strategy Search.pdf]

MEDLINE (by PUBMED) search strategy

Search strategy
1. child
2. children
3. adolescent
4. teens
5. teenagers
6. human immunodeficiency virus
7. HIV
8. Acquired Immune Deficiency Syndrome Virus
9. physical fitness
10. fitness tracked
11. physical endurance
12. muscle strength
13. muscular endurance
14. physical conditioning
15. aerobic capacity
16. cardiorespiratory fitness
17. flexibility
18. pliability
19. #1 OR #2 OR #3 OR #4 OR #5
20. #6 OR #7 OR #8
21. #9 OR #10 OR #11 OR #12 OR #13 OR #14 OR #15 OR #16 OR #17 OR #18
22. #19 AND #20 AND #21
23. #21 AND #19
