# Supplementary material for: Comparison of Muscle Strength, Aerobic Capacity and Body Composition between Healthy Adolescents and Those Living with HIV: A Systematic Review and Meta-Analysis
Source: Int J Environ Res Public Health. 2021 May 26;18(11):5675. doi: 10.3390/ijerph18115675 (PMC8198095; doi:10.3390/ijerph18115675)
Supplement: Supplementary file 1 [file ijerph-18-05675-s001.zip › Supplement 2-Quality of Studies.pdf]

Criterias / Questions	Study				
	Cade et al., 2002	Lima et al., 2017	Martins et al., 2017	Ramos et al., 2012	Somarriba et al., 2013
Q1=Research question	Yes	Yes	Yes	Yes	Yes
Q2 =Study population	Yes	Yes	Yes	Yes	Yes
Q3= Study Population (elegíveis era de pelo menos 50%)	No	No	No	No	No
Q4=population/eligibility criteria	Yes	Yes	Yes	Yes	Yes
Q5=Sample size	Yes	No	No	No	No
**Selection bias (Maximum = 5)**	**4**	**3**	**3**	**3**	**3**
Q6= Exposure assessed prior to outcome measurement	No	No	No	No	No
Q7= Sufficient timeframe to see an effect	No	No	No	No	No
Q8= Different levels of the exposure of interest	No	No	No	No	No
Q9= Exposure measures and assessment	Yes	Yes	Yes	Yes	Yes
Q10= Repeated exposure assessment	No	No	No	No	No
Q11= Outcome measures	Yes	Yes	Yes	Yes	Yes
Q12= Blinding of outcome assessors	No	No	No	No	No
**Measurement and outcome bias (maximum = 7)**	**2**	**2**	**2**	**2**	**2**
Q13= Follow-up rate	No	Yes	No	No	No
Q14 = Statistical analyses	Yes	No	No	No	No
**Data presentation (Maximum = 7)**	**1**	**0**	**2**	**0**	**0**
**Total score**	**7**	**6**	**5**	**5**	**5**
