# Supplementary material for: Comparison of Muscle Strength, Aerobic Capacity and Body Composition between Healthy Adolescents and Those Living with HIV: A Systematic Review and Meta-Analysis
Source: Int J Environ Res Public Health. 2021 May 26;18(11):5675. doi: 10.3390/ijerph18115675 (PMC8198095; doi:10.3390/ijerph18115675)
Supplement: Supplementary file 1 [file ijerph-18-05675-s001.zip › Table S1.pdf]

Supplementary material

Table S1: Studies excluded.

Authors, year	Decision
Adams et al., 2012	Wrong design (Experiment studies)
Braz et al., 2014	No comparator group (no group control)
Botros et al., 2012	Wrong design (review)
Burke et al., 2001	No comparator group (no group control)
Campo et al, 2014	Wrong population (no Children)
Campbell et al., 2014	No comparator group (no group control)
Crawford et al, 2013	Wrong population (no Children)
Cade et al, 2003	Wrong population (no Children)
Coodley et al., 1997	Wrong design (Experiment studies)
Coura et al., 2011	Wrong design (review)
Chisati and Vasseljen, 2015	Wrong population (no Children)
Dellar et al., 2014	Wrong design (Experiment studies)
Evans et al., 2002	Wrong design (Experiment studies)
Fabiano et al., 2013	No comparator group (no group control)
Farinatti et al., 2010	Wrong population (no Children)
Gellato et al., 2019	No comparator group (no group control)
Giordano et al., 2018	Wrong design (longitudinal studies)
Gomes-Neto et al., 2018	Wrong design (review)
Jemmont et al., 2011	No comparator group (no group control)
Lox et al., 1996	Wrong design (review)
Macdonald et al, 2017	Wrong population (no Children)
Martins et al., 2019	No comparator group (no group control)
Mhariwa, 2015	Wrong population (no Children)
Malita et al., 2005	Wrong design (review)
Malete et al., 2017	Wrong Outcomes

Matsudo et al., 2015	No comparator group (no group control)
McDermott et al., 2017	Wrong design (Experiment studies)
Miller et al., 2010	No comparator group (no group control)
Olsen et al, 2015	Wrong population (no Children)
Oliveira et al, 2017	Wrong population (no Children)
Oursler et al, 2006	Wrong population (no Children)
Patil et al, 2017	Wrong population (no Children)
Pavlinac et al., 2014	No comparator group (no group control)
Penner et al., 2018	Wrong design (review)
Pothoff et al, 1994	Wrong population (no Children)
Puthanakit et al., 2013	No comparator group (no group control)
Raso et al, 2016	Wrong population (no Children)
Rhoads et al., 2011	Wrong Outcomes
Ortmeyer et al, 2016	Wrong population (no Children)
Ousler et al., 2006	Wrong population (no Children)
Silva et al, 2015	Wrong population (no Children)
Silva et al, 2014	Wrong population (no Children)
Sonaglio et al., 2011	No comparator group (no group control)
Tanaka et al., 2015	No comparator group (no group control)
Trost et al., 1996	Wrong Outcomes
Zanetti et al., 2016	Wrong design (Experiment studies)
Wallet et al, 2015	Wrong population (no Children)
Wenner	No comparator group (no group control)
Weggheleire et al., 2012	Wrong design (review)
